# Quality of Life and Its Associations with Religiosity and Religious Coping among Outpatients with Psychosis in Singapore

**DOI:** 10.3390/ijerph18137200

**Published:** 2021-07-05

**Authors:** Kumarasan Roystonn, Laxman Cetty, Anitha Jeyagurunathan, Fiona Devi, Edimansyah Abdin, Soo Teng Tan, Charmaine Tang, Swapna Verma, Mythily Subramaniam

**Affiliations:** Institute of Mental Health, Singapore 539747, Singapore; Laxman_Cetty@imh.com.sg (L.C.); Anitha_Jeyagurunathan@imh.com.sg (A.J.); Fiona_Devi_Siva_Kumar@imh.com.sg (F.D.); Edimansyah_Abdin@imh.com.sg (E.A.); Tan_Soo_Teng@imh.com.sg (S.T.T.); Charmaine_YZ_Tang@imh.com.sg (C.T.); Swapna_Verma@imh.com.sg (S.V.); Mythily@imh.com.sg (M.S.)

**Keywords:** religious coping, religiosity, quality of life, psychosis, Asia, Singapore

## Abstract

This cross-sectional study investigated the relationship of religiosity, the use of positive and negative religious coping methods, and quality of life (QOL) among 364 outpatients with psychosis in Singapore. Positive religious coping was significantly associated with better scores on physical (β = 0.51, *p* = 0.02) and psychological (β = 0.64, *p* = 0.01) QOL domains in the regression model. Negative religious coping was related to worse QOL in all four domains: physical (β = −0.44, *p* = 0.03), psychological (β = −0.76, *p* < 0.01), social (β = −0.54, *p* = 0.03), and environment (β = −0.65, *p* < 0.01). Increased participation in organizational religious activities was positively associated with higher QOL for psychological (β = 2.47, *p* < 0.01), social relationships (β = 2.66, *p* = 0.01), and environment (β = 2.09, *p* = 0.01) domains. Interestingly, those with no religious affiliation were found with higher scores in the QOL domain for social relationships (β = 4.59, *p* = 0.02). Religious coping plays an important role for the QOL of outpatients with psychosis. Greater awareness of the importance of religion in this population may improve cultural competence in treatment. Individuals with psychosis may benefit from greater community support and collaboration between clinical and religious community-based organizations to improve social integration and QOL.

## 1. Introduction

Psychotic illnesses are among the most debilitating of mental health disorders [1,2,3]. Recently, mental health service delivery has begun to adopt a recovery paradigm [4,5], embracing a shift toward rehabilitation and a holistic approach which considers the various aspects of wellbeing and functioning [6]. Quality of life (QOL) has, thus, increasingly gained attention as a critical outcome measure of psychiatric diagnoses, particularly among those with psychosis [7].

QOL is a multidimensional concept involving multiple aspects of individuals’ physical, emotional, environmental, and social wellbeing [8]. Researchers have increasingly witnessed that a person’s spiritual or religious experiences also contribute to their quality of life [9,10]. Religion is an important coping strategy when faced with stressful life experiences [11]. Religion may play a role in the long-term adjustment, maintain one’s self-esteem, deliver emotional comfort and hope, and provide a sense of meaning and purpose [12]. While religious coping has been consistently found to be an important coping resource affecting QOL, the research has mainly been in the context of chronic and life-threatening illnesses such as cancer [13], hemodialysis [14], epilepsy [15], and human immunodeficiency viruses [16].

Religion has a complex role in patients with psychosis. Previous research has shown that individuals with psychosis give great importance to spirituality/religion in their lives and report frequent participation in personal and organized religious activities [17]. Religion offers a form of positive coping such as prayer, reinforcing a belief in a benevolent higher power, as well as a sense of connectedness with a religious community during such mental health crises [18,19]. However, not all religious coping strategies may be useful and adaptive for the individual [20]. Negative religious coping attributes life situations to a punishing God and feelings of abandonment from God, reflecting a belief in a hostile higher power and a sense of disconnectedness from the religious community [21,22]. In a cross-sectional study on religious coping among persons with schizophrenia, the researchers found that patients reported religion as important in coping, and the extent to which they depend on it for coping with their illness correlated with fewer symptoms, improved social functioning, and reduced suicide attempts. On the other hand, negative religious coping was found inversely associated with these same outcomes [23]. Beyond cross-sectional studies, prospective longitudinal studies also provide some evidence of religion being linked to clinical and functional outcomes. In India, a 2-year follow-up study found that greater frequency of participation in religious practices predicted better clinical and functional outcomes [24]. However, less attention has been paid to determining the type of religious coping, positive or negative, which may differentially affect QOL outcomes in individuals with psychosis. The relationship between religious coping and QOL of patients with psychotic disorders has, to our best knowledge, not been examined comprehensively with both positive and negative dimensions, especially in a multiethnic, multicultural setting. The purpose of this study was to determine the relationship between religious coping and quality-of-life dimensions among individuals with a broad range of psychotic illnesses. We hypothesized that positive religious coping is related to better QOL and that negative religious coping is associated with worse QOL in all dimensions among individuals living with psychosis in the community.

## 2. Materials and Methods

### 2.1. Study Design and Population

This cross-sectional study collected data from participants who were outpatients seeking treatment at a public psychiatric tertiary hospital. Participation criteria included fulfilling DSM-IV criteria of psychosis-related disorders (excluding substance-induced psychosis), no developmental disability, literacy in English, an age between 21 and 65, and being a citizen or permanent resident of Singapore. A person’s ability to complete the self-report study questionnaire was determined by the researcher on a case-by-case basis. Data collection took place during January 2018 and April 2019.

Attending clinicians referred eligible participants who were interested to the study team members. Recruitment flyers were also posted in the waiting areas of the outpatient clinic. Participants completed the study questionnaire at the clinic. The study data were later recorded using the electronic data collection software, REDCap (Research Electronic Data Capture). Ethical approval for the study was obtained from the National Healthcare Group, Domain Specific Review Board, and written informed consent was obtained from all participants prior to the commencement of study procedures.

### 2.2. Measures

#### 2.2.1. World Health Organization Quality of Life BREF (WHOQOL-BREF)

The primary outcome measure, QOL, was measured with the World Health Organization Quality of Life BREF (WHOQOL-BREF) [25]. This questionnaire has been validated among individuals living with psychosis [26,27]. WHOQOL-BREF assesses subjective quality of life within a profile containing four main domains of an individual’s perception within the last 2 weeks: physical health, psychological health, social relationships, and relationship with one’s environment. Two items assess overall quality of life and satisfaction with general health. Transformed and scaled scores on each WHOQOL-BREF domain range from 0 to 100 (33), with higher scores indicating a higher quality of life. In this study, the Cronbach α values were as follows: physical (0.72), psychological (0.84), social relationships (0.69), and environment (0.86).

#### 2.2.2. Brief Religious Coping Scale (B-RCOPE)

Positive and negative religious coping was evaluated with the 14-item B-RCOPE. The B-RCOPE has demonstrated good concurrent validity [28]. A positive coping pattern consists of religious forgiveness, seeking spiritual support, collaborative religious coping, spiritual connection, religious purification, and benevolent religious reappraisal. A negative pattern consists of spiritual discontent, appraisal of God as punishing, interpersonal religious discontent, appraisal of demonic powers, and reappraisal of God’s powers. Each item is measured from 0 (“not at all”) to 3 (“a great deal”). Scores on the respective subscales of B-RCOPE range from 0 to 21, with higher scores indicating increased positive or increased negative religious coping. As this study recruited participants across the range of the religious spectrum, the version recommended with non-Western samples was used, wherein the phrases “church” and “God” were replaced with “religious community” and “God/Higher Power”, respectively, in order to assess other religious orientations that do not center around “God” (e.g., Hinduism, Buddhism) [29]. The positive religious coping scale had an α of 0.94, while the negative religious coping scale had an α of 0.86.

#### 2.2.3. Duke University Religion Index (DUREL)

Religious commitment was examined with the five-item DUREL [30], which assesses major dimensions of organizational and non-organizational religiosity, along with intrinsic religiosity. Organizational religiosity was determined by the frequency of attendance at religious services, and non-organizational religiosity was determined by the frequency of participation in private religious activities such as prayer, meditation, or Bible study. The organizational and non-organizational items were rated on a six-point Likert scale. Intrinsic religiosity comprised three items, rated on a five-point Likert scale, and determined whether one has experienced the presence of the divine, allowed religious beliefs to guide their approach to life, and if religion influences other areas of their life.

#### 2.2.4. Other Variables

Covariates used in this study included sociodemographic, health, and clinical factors, known to independently affect QOL outcomes in persons with psychotic illnesses [31,32]. Information on primary psychiatric diagnosis, illness duration, and age of onset of psychosis were obtained from medical records, and self-reported sociodemographic details including age, ethnicity, gender, marital status, highest educational qualifications, and religious affiliation were included in the data analysis. Participants were also asked to indicate the extent to which they considered religion to be important (1 = of no importance, 2 = of some importance, 3 = important, 4 = very important, 5 = essential). This was regrouped into a dichotomous variable of “not important” (of no importance and of some importance), and “important” (important, very important, and essential) for regression analysis.

### 2.3. Statistical Analyses

The means, standard deviations, and ranges of scores for continuous variables and the frequencies and percentages for categorical variables were calculated to provide a description of the sample. The study included two predictors of interest (positive and negative religious coping) and four QOL dependent variables (physical health, psychological health, social relationships, and environment). The relationships between sociodemographic variables and the key variables in the study (religious coping, religiosity, and QOL domains) were examined. Multiple linear regression analyses were performed to examine the relationship of positive and negative religious coping in each QOL dimension, controlling for key sociodemographic and clinical predictors of QOL. All tests utilized a *p* < 0.05 criterion for statistical significance. All descriptive statistical analyses were conducted using SPSS (IBM, Armonk, NY, USA). Multivariate linear regression analyses were conducted with RStudio statistical software (RStudio PBC, Boston, MA, USA).

## 3. Results

### 3.1. Participant Characteristics

The results are presented in Table 1. In general, the 364 patients sampled (53.8% female) were mostly Chinese (69.2%), single (74.7%), and young (mean age, 35.2 years), with at least a secondary-school education (97.5%). Among the patients, the mean duration of living with a psychotic illness was 8.4 (SD: 8.9) years. The majority (69.2 %) believed religion was compatible with psychiatric treatment. There was some religious diversity; 84.6% were either Christian (34.1%), Muslim (20.1%), Buddhist/Taoist (25.8%), or belonged to other religions (4.6%), while 14% of them practiced no religion. Religion was “important”, “very important”, or “essential” to 68.6% of the patients in coping with illness, whereas 15.7% said it was “of some importance”, and 15.4% said it was not important.

### 3.2. Religious Coping, Religious Commitment, and QOL Domains

In Table 2, scores on the RCOPE were 18.5 ± 6.9 for positive religious coping and 12.3 ± 5.3 for negative religious coping. Patients reported mean scores of 3.5 (of 6) for organizational religious activity, 4.1 (of 6) for non-organizational religious activity, and 7.6 (of 15) for intrinsic religiosity scores on the DUREL. According to the transformed scores from the WHOQOL-BREF scale, patients reported 64.1 (16.0) in the physical, 56.1 (19.2) in the psychological, 58.8 (20.0) in the social, and 62.4 (17.5) in the environment domains of QOL. Patients reported a mean score of 3.55 (range: 1–5) and a standard deviation of 0.9 for the overall QOL. Table 3 describes the mean scores for religious commitment, religious coping pattern, and the respective WHOQOL-100 domains by religious affiliation. On the DUREL, the highest mean scores were observed among the Christian participants for organizational religious activity (4.3 ± 1.4) and intrinsic religiosity (11.3 ± 2.8). According to Table 3, participants who were Muslim reported the greatest use of positive religious coping (23.3 ± 5.4) and negative religious coping (14.1 ± 6.1).

### 3.3. Relationship between Religious Coping and QOL

Table 4 displays the multiple linear regression model of positive and negative religious coping in the QOL domains after controlling for key sociodemographic and clinical variables including age, gender, marital status, education, diagnosis, religious affiliation, importance of religion in illness coping, and duration of illness. A greater use of positive religious coping was associated with better scores in the physical (*β* = 0.51, *p* = 0.02) and psychological (*β* = 0.64, *p* = 0.01) domains of QOL. Negative religious coping was related to worse QOL in all four domains: physical (*β* = −0.44, *p* = 0.03), psychological (*β* = −0.76, *p* < 0.01), social (*β* = −0.54, *p* = 0.03), and environment (*β* = −0.65, *p* < 0.01) (Table 4).

### 3.4. Relationship between Religiosity and QOL

In addition, participants who reported religion as important in coping (*β* = 8.09, *p* = 0.01) were related to better scores in the psychological dimension of QOL (see Table 4). Participation in organizational religious activities on the DUREL was positively associated with higher QOL for psychological (*β* = 2.47, *p* = 0.01), social (*β* = 2.66, *p* = 0.01), and environment (*β* = 2.09, *p* = 0.01) domains. Participants with no religious affiliation were associated with higher scores (*β* = 4.59, *p* = 0.02) in the QOL domain for social relationships compared to those of Christian religious affiliation.

## 4. Discussion

The purpose of this article was to examine how the use of positive and negative religious coping among patients with psychosis was related to the multidimensional components of QOL. The findings indicate that greater use of positive religious coping was related to better scores in the physical and psychological domains of QOL, and greater use of negative religious coping was associated with lower scores for QOL across all domains. Overall, our findings suggest that both positive and negative religious coping are associated with QOL in outpatients living with psychosis and, thus, religion may have significant influences on the individual’s wellbeing and recovery from illness.

Although in the recent decade there has been a slight increase in consensus on addressing the topic of religion in clinical practice, much of the controversy lies in whether healthcare professionals should encourage religious activities in patients with mental illness [33]. Furthermore, there is a modest but growing trend toward religious non-affiliation documented worldwide [34]. While one in five Singaporean residents reported no religious affiliation or “none” according to the recent census of population [35], it is evident from the current study that, for patients with psychosis in Singapore, the use of religious coping is prevalent and of high importance to one’s subjective quality of life. Moreover, our findings show that an examination of religious coping, particularly negative religious coping, might be crucial to better understand patients who experience poorer QOL.

In this study, patients with psychosis reported similarly high levels of use of both positive and negative religious coping. Research has previously reported how religion may intervene in psychiatric treatment [20,21,22,23]. It is then crucial to attend to such negative coping methods concerning, for example, anger at God and feelings of abandonment or punishment by God, so as to increase the likelihood of improving QOL in multiple dimensions and reducing the propensity for negative health and clinical outcomes. Conversely, for those who tend to utilize positive religious coping, it would be just as important to ensure that related resources and community-based religious support are made more available so that these patients can maintain their quality of life and wellbeing, with greater improvement in the environment and social domains of their QOL.

Our study results show that higher scores for organizational religiosity and intrinsic religiosity were observed among participants who were Christian, while participants who were Muslim reported far greater use of both positive and negative religious coping. Our study findings also revealed that those who reported greater frequency of participation in organized religious activity were associated with better scores in the psychological, social, and environment domains of QOL. These findings are consistent with recent research exploring religiosity and religious coping among Christian and Muslim residents of the United Arab Emirates (UAE) during the COVID-19 pandemic [36]. These authors found that the Muslim cohort reported higher levels of religious coping compared to their Christian counterparts, even though the latter showed greater frequency of participation in religious activities. It might be posited that these findings reflect the nature of different community practices and expectations in Islam and Christianity. However, it should be noted that the UAE and Singapore are still rather different in terms of Islamic activities and position in the community. The UAE is a Muslim country that holds many of the community-based requirements and practices of other Middle Eastern countries [37], while Singapore possesses much greater diversity. Nevertheless, our findings suggest the importance of religious community activities and support from a religious community for some individuals with psychosis living in the community. Mental health initiatives by religious organizations and community rehabilitation programs for religious persons with psychosis may benefit from including a focus on positive religious coping strategies to lead to beneficial adaptation and reappraisal of the illness and related stressors.

Religious organizations contribute significantly to the integration of the community, thereby enhancing social relationships and support available for patients who use religion to cope with their psychotic illness and related stressors. Interestingly, those with no religious affiliation in this psychiatric sample were found to have significantly better QOL in their social relationships compared with those of Christian religious affiliation. We propose a plausible explanation for this phenomenon. The social domain of QOL broadly covered several aspects including personal satisfaction with close relationships, satisfaction with one’s sex life, and support from close friends. On the other hand, organized religious activity on the DUREL tends to be centered on one’s participation in cultural and religious community activities, which may not necessarily reflect the level of support one receives. Hence, individuals of Christian religious affiliation in our sample appeared to have lower scores in the social QOL domain, despite having significantly higher levels of organized religious activity compared to others. We posit that these individuals could have also been compensating with greater involvement in organized religious activity, and future longitudinal studies are necessary to better understand this phenomenon. Our findings could also suggest that individuals with psychosis are not necessarily at a disadvantage if they do not use religion for coping. Some scholars suggest that one’s subjective wellbeing and quality of life outcomes may be mediated to some extent by perceived levels of social support available to the individual [38]. The same mechanisms may be at work here, and future work is necessary to explore these possibilities with similar multicultural samples of patients with psychotic disorders living in the community.

### Strengths and Limitations

First, the exploratory and cross-sectional nature of the data does not allow for conclusions about causality to be made in either direction. Thus, although it is likely that religious coping methods influence QOL in particular ways, it is equally possible that patients who experience better QOL turn to religious resources for coping with their psychotic illness. Although the data in the current study are cross-sectional, prior longitudinal research has reported to some degree that religious coping use has effects on QOL domains among individuals with chronic medical or psychosis-related conditions such as schizophrenia and schizoaffective disorder [22,23]. All of this suggests that the study findings may be considered for the implications on the QOL of individuals with psychotic disorder, especially for patients who endorse greater use of positive and negative religious coping.

Second, our findings must be considered preliminary and must be replicated in future studies. For example, the DUREL was adapted to include the measure of religiosity and spirituality of Eastern religious traditions (e.g., Hinduism or Buddhism). It is quite possible that the assessment of some religious and spiritual practices of the patients may not be a true reflection of their religiosity [30]. Further studies in this population are needed to confirm or refute our findings and to validate the use of the adapted DUREL in this population. Moreover, research should consider other potential factors such as the duration of religious affiliation. Third, the results must be considered in the context of the population examined. The study participants were receiving outpatient treatment and living in the community. It could be that they are less distressed and may have been more likely to report a greater use of positive religious coping than negative religious coping. Moreover, this study was limited to a multicultural, multireligious sample. Thus, future research should examine these effects in other similar samples of patients with psychosis with greater distress, as well as among in-patient or mono-religious populations, for comparative research.

Despite these limitations, this study is one of the first to examine the role of positive and negative religious coping in relation to the multiple domains of QOL among community-dwelling individuals with psychosis in Singapore. A major strength of the study is that the data reflect the multicultural perspectives of outpatients with psychosis and comprehensively examine religiosity and both positive and negative aspects of religious coping. Additionally, given that the manifestation of religion can vary across cultures, the study methodology demonstrates greater sensitivity to the terms related to religion, spirituality, and its practice, using the Brief-RCOPE version that replaces terms such as “church” and “God” with “religious community” and “God/Higher Power” [29], thus increasing the external validity of our study findings.

## 5. Conclusions

The study findings revealed that religious coping is significantly associated with several domains of QOL. Although the scope of the study was limited to Singapore, the results can be generalized to other cross-cultural and religiously diversified pluralistic societies. This underscores the importance of religion in this population and suggests the need for improving cultural competencies in clinical treatment and care services. Spiritual care should be incorporated in treatment plans to benefit recovery, reduce relapse, and improve the quality of life of mental health patients. The results further indicate that outpatients living with psychotic disorders may benefit from greater support in the community, and future research should explore strengthening the collaboration between clinical and religious community-based organizations to contribute to better symptom relief, as well as improve the social integration and quality of life of individuals with psychosis.

## Figures and Tables

**Table 1:** Sociodemographic and clinical variables (*n* = 364).

		Mean/Frequency	Std. Deviation/Percentage
Age (years)		35.2	10.8
Age at onset (years)		26.7	7.9
Illness duration (years)		8.4	8.9
Gender	Male	168	46.2
	Female	196	53.8
Marital status	Single	272	74.7
	Married	71	19.5
	Divorced/separated/widowed	21	5.8
Education	Primary and below	9	2.5
	Secondary	93	25.5
	Pre-tertiary	170	46.7
	Tertiary and above	92	25.3
Religious affiliation	Christianity	124	34.1
	Buddhism and Taoism	94	25.8
	Islam	73	20.1
	Hinduism	15	4.1
	Sikhism	2	0.5
	No affiliation	51	14.0
Importance of religion in illness coping	No importance	56	15.4
	Some importance	57	15.7
	Important	78	21.4
	Very important	75	20.6
	Essential	97	26.6

There were five cases of missing information for religious affiliation and one case of missing information for importance of religion in illness coping.

**Table 2:** Religious commitment, religious coping, and WHOQOL-100 domain scores.

	Mean	Standard Deviation
Religious coping		
Positive	18.5	6.9
Negative	12.3	5.3
Religious commitment		
Organizational religious activity	3.5	1.4
Non-organizational religious activity	4.1	1.8
Intrinsic religiosity	7.6	3.4
WHOQOL-100		
Physical	64.1	16.0
Psychological	56.1	19.2
Social relationships	58.8	20.0
Environment	62.4	17.5
Overall QOL	3.55	0.9

**Table 3:** Religious coping, religious commitment, and WHOQOL-100 domain scores by religious affiliation.

	Religious Affiliation					
Mean (SD)	Christianity	Buddhism	Islam	Hindu	Sikh	* *p*-Value
Religious commitment						<0.01
Organizational religious activity	4.3 (1.4)	3.0 (1.1)	3.7 (1.5)	3.5 (1.2)	4.0 (1.4)	
Non-organizational religious activity	3.5 (1.8)	2.3 (1.4)	3.6 (1.7)	2.9 (1.9)	2.0 (1.4)	
Intrinsic religiosity	11.5 (2.8)	9.5 (3.0)	11.6 (3.2)	10.9 (2.6)	8.5 (2.1)	
Religious coping						<0.01
Positive	20.4 (5.7)	15.6 (6.2)	23.3 (5.4)	17.9 (4.5)	18.5 (9.2)	
Negative	12.9 (5.2)	11.2 (4.7)	14.1 (6.1)	10.4 (3.7)	8.0 (1.4)	
WHOQOL-100						<0.01
Physical	64.8 (15.8)	65.0 (15.5)	60.6 (18.6)	70.5 (12.8)	59.5 (30.4)	
Psychological	57.0 (18.1)	55.2 (18.8)	56.7 (21.7)	60.9 (22.5)	59.5 (48.8)	
Social relationships	57.5 (19.9)	58.4 (17.3)	60.3 (23.5)	63.0 (26.9)	56.5 (53.0)	
Environmental	63.5 (18.0)	62.8 (15.8)	59.2 (20.6)	61.1 (16.2)	72.0 (22.6)	

* Derived from ANOVA test of statistically significant differences between independent groups.

**Table 4:** Multiple linear regression model of religiosity and religious coping on QOL domains.

Variables		Physical QOL				Psychological QOL				Social QOL				Environment QOL			
		β	SE	t	*p*	β	SE	t	*p*	β	SE	t	*p*	β	SE	t	*p*
Religious coping	Positive	0.51	0.22	2.27	**0.02**	0.64	0.26	2.45	**0.01**	0.17	0.28	0.61	0.54	0.45	0.24	1.91	0.06
	Negative	−0.44	0.20	−2.25	**0.03**	−0.76	0.23	−3.29	**<0.01**	−0.54	0.24	−2.21	**0.03**	−0.65	0.21	−3.13	**<0.01**
Importance of religion	Not important	Ref				Ref				Ref				Ref			
	Important	1.96	2.48	0.79	0.43	8.09	2.91	2.78	**0.01**	4.64	3.08	1.51	0.13	4.36	2.62	1.66	0.10
Religious activity	Organized RA	1.05	0.78	1.35	0.18	2.47	0.91	2.72	**0.01**	2.66	0.96	2.76	**0.01**	2.09	0.82	2.55	**0.01**
	Non-organized RA	−0.45	0.64	−0.71	0.48	−1.09	0.75	−1.46	0.15	−1.33	0.79	−1.67	0.10	−0.45	0.68	−0.67	0.51
	Intrinsic religiosity	−0.42	0.39	−1.07	0.28	−0.42	0.46	−0.92	0.36	0.85	0.49	1.75	0.08	0.13	0.41	0.31	0.76
Religious affiliation	Christianity	Ref				Ref				Ref				Ref			
	Buddhism	3.03	2.47	1.23	0.22	2.93	2.89	1.02	0.31	5.20	3.06	1.70	0.09	3.40	2.61	1.30	0.19
	Islam	−2.28	2.67	−0.85	0.39	−0.23	3.12	−0.07	0.94	5.08	3.32	1.53	0.12	−3.03	2.82	−1.08	0.28
	Hindu	7.24	4.55	1.59	0.11	3.47	5.33	0.65	0.51	4.24	5.66	0.75	0.45	−2.15	4.81	−0.45	0.66
	Sikh	−5.64	11.74	−0.48	0.63	3.03	13.75	0.22	0.83	−0.70	14.59	−0.05	0.96	11.04	12.42	0.89	0.37
	No affiliation	−0.89	3.70	−0.24	0.81	4.12	4.33	0.95	0.34	10.38	4.59	2.26	**0.02**	6.87	3.91	1.76	0.08

Adjusted for age, gender, marital status, education, diagnosis, and duration of illness. Bold print highlights statistically significant *p*-values at *p* < 0.05.

## Data Availability

The datasets and material used and/or analyzed during the current study are available from the corresponding author upon reasonable request.
